# Polymorphisms in the multidrug-resistance 1 gene related to glucocorticoid response in rheumatoid arthritis treatment

**DOI:** 10.1007/s00296-017-3653-1

**Published:** 2017-01-28

**Authors:** Bart V. J. Cuppen, Katerina Pardali, Maarten C. Kraan, Anne C. A. Marijnissen, Linda Yrlid, Marita Olsson, Johannes W. J. Bijlsma, Floris P. J. G. Lafeber, Ruth D. E. Fritsch-Stork

**Affiliations:** 1 Rheumatology and Clinical Immunology, University Medical Center Utrecht, F02.127, 3508 GA Utrecht, The Netherlands; 2 Respiratory and Inflammation iMed, AstraZeneca, Pepparedsleden 1, 431 83 Mölndal, Sweden; 3 1st Medical Department and Ludwig Boltzmann Institute of Osteology at the Hanusch Hospital of WGKK and AUVA Trauma Centre Meidling, Hanusch Hospital, Vienna, Austria; 4 Sigmund Freud University, Vienna, Austria

**Keywords:** Multidrug resistance, MDR1, Rheumatoid Arthritis, Glucocorticoids, Polymorphisms

## Abstract

A substantial proportion of rheumatoid arthritis (RA)-patients experience an insufficient response to glucocorticoids, an important therapeutic agent in RA. The multidrug-resistance 1 (MDR1) gene product P-glycoprotein (P-gp) is an efflux pump that actively transports substrates, such as glucocorticoids, out of the cell. We investigated if the variation in response might be explained by single-nucleotide polymorphisms (SNPs) in the MDR1 gene. RA-patients treated with intravenous methylprednisolone pulses (*n* = 18) or oral prednisone/prednisolone (*n* = 22) were included in a prospective cohort, and clinical response was measured after 5 and 30 days, respectively. The C1236T, G2677A/T, and C3435T SNPs were determined, and the functionality of P-gp was assessed by flow cytometry (Rhodamine efflux assay). Carriage of the G2677A/T SNP was significantly associated with response (OR = 6.18, *p* = 0.035), the other SNPs showed trends. Stratified for received treatment, the effect was only present in methylprednisolone treated patients. Mutant allele carriage significantly decreased functionality of P-gp in B cells, though had a smaller impact in other PBMC subtypes. Carriage of a MDR1 SNP was related to a response to methylprednisolone in this study, which his suggests that RA-patients carrying wild-type alleles might benefit from P-gp inhibition or administration of glucocorticoid analogues that are non-P-gp substrates.

**Electronic supplementary material:**

The online version of this article (doi:10.1007/s00296-017-3653-1) contains supplementary material, which is available to authorized users.

## Introduction

Rheumatoid arthritis (RA) is a chronic, disabling disease that mainly affects the synovial joints. Glucocorticoids (GC) compose a class of drugs that have an important place in RA treatment [1]; however, a substantial proportion of patients experiences insufficient response.

The multidrug-resistance 1 (MDR1) gene product, the P-glycoprotein (P-gp), is an efflux pump that actively transports substrates, such as drugs, out of the cell. The ability to regulate intracellular substrate concentration depends on both the expression and the functionality (i.e., recognition of substrates and transport effectiveness) of P-gp [2]. In treatment refractory RA, P-gp overexpression in lymphocytes is believed to play a substantial role, as it has been shown that overexpression reduces intracellular concentrations of immunosuppressants that are P-gp substrates [3]. Single-nucleotide polymorphisms (SNPs) have been linked to P-gp activity and are suspected to influence inter-individual variation in response to certain treatments [4, 5]. Because GCs are known P-gp substrates [6, 7], differences in GC response might be explained by carriage of polymorphisms in the MDR1 gene. We, therefore, explored the possibility that carriers of mutant alleles in the MDR1 gene efflux GCs less effectively, and subsequently experience a better clinical response.

## Methods

### Patients and sample collection

RA-patients treated with GCs at our department of Rheumatology were included in a prospective cohort. Patients eligible for intravenous (IV) GC pulse treatment according to their treating rheumatologist were given three doses of 1000 mg IV methylprednisolone on alternate days, as this approach has been proven to be quick, effective (in short and long term), and safe [8–10]. RA-patients with an indication for oral GC treatment were treated with prednisone or prednisolone doses between 7.5 and 10 mg for at least 1 month. Based on our clinical experience and the previous research on IV pulse treatment [8], the optimal time point to measure (maximum) treatment effect was considered 5 days after start. Treatment response in orally treated patients was assessed at day 30 which was considered sufficient as a response as early as two weeks is already a strong indicator of the long-term clinical outcome [11]. DAS28 response was evaluated according to the European league against Rheumatism (EULAR) response criteria [12]. EULAR good response, i.e., a DAS ≥ 1.2 and DAS28 < 3.2, was compared to the combination of moderate and non-response considering the intensity of the treatment, especially in the IV arm.

Whole blood was collected in Li-Heparin tubes. Peripheral blood mononuclear cells (PBMCs) were isolated and stored in liquid nitrogen until use. The study was approved by the medical ethics committee of the University Medical Center Utrecht and was conducted in accordance with the Helsinki Declaration. All patients signed informed consent.

### Genotyping of MDR1

Genomic DNA was extracted from 10^5^ PBMCs using QIAamp DNA blood mini kit (Cat. No 51104, Quigen, Hilden, Germany) according to manufacturer’s instructions. C1236T (rs1128503), G2677A/T (rs2032582), and C3435T (rs1045642) SNPs were genotyped using TaqMan allelic discrimination assay technology (Applied Biosystems, Foster City, California, USA) on a QuantStudio 7 Flex Real-Time PCR System (Thermo Fisher Scientific Inc, Foster City, California, USA), according to the manufacturer’s instructions. The analyses were run in duplicate. The mutant allele frequency of the study cohort (observed frequency) was compared with the Central European occurrence (expected allele frequency, derived from 1000 Genomes catalog [13]).

### Functionality of P-gp

The functionality of P-gp was assessed by flow cytometry, using a Rhodamine efflux assay. 4.0 × 10^5^ cryo-preserved PBMCs were incubated with Rhodamine 123 (Sigma–Aldrich, St. Louis, USA) at a final concentration of 1 μg/ml for 30 min at 4 °C and washed twice. After dividing cells in two wells, they were incubated in the presence (control) or absence of the MDR1-inhibitor Elacridar (Astrazeneca, Mölndal, Sweden). The cells were stained with antibodies to determine subsets of PBMCs: CD3, CD4, CD8, CD14, CD16, CD19, CD196, and CD56 (all BD Bioscience, San Jose, USA). Flow cytometry was performed on a four laser LSR Fortessa (BD Bioscience, San Jose, USA) and samples were analyzed using FlowJo (Tree Star Inc. Ashland, USA). Only baseline samples (i.e., before start of therapy) with a clear FSC vs SSC profile containing living PBMCs were used for data analysis. As additive test for viability and proper labeling with Rhodamine 123, all samples were excluded that had >5% low Rhodamine 123 containing cells in the elacridar treated sample. After gating of living cells in the FSC vs SSC plot, duplicates and cell-aggregates were removed using FSC-A vs FSC-H plot. The frequency of cells effluxing Rhodamine 123 in the uninhibited samples was used as a value for transport functionality.

### Statistical analysis

Associations between each SNP and patient’s response were analyzed using logistic regression models, in which each investigated SNP was tested while adjusted for treatment received (IV/oral). SNPs were first analyzed in a dominant model in which the SNP was coded dichotomously (wild-type/mutant allele carrier) and subsequently in an additive model [linear covariate coded: zero mutant alleles (wild-type)/one mutant allele (heterozygous)/two mutant alleles (homozygous mutant)]. All analyses were performed in SPSS 21 (IBM Corp., Armonk, NY).

## Results

Baseline characteristics of patients treated with IV methylprednisolone (*n* = 18) and oral prednisone/prednisolone (*n* = 22) are shown in Table 1. There were no differences between the cohorts, apart from a higher DAS28 at baseline in line with the difference in clinical indication for the two treatment regimens.

**Table 1:** Baseline characteristics for intravenous and oral GC treated cohorts

Item	IV cohort (*n* = 18)	Oral cohort (*n* = 22)	*p* value
Female gender, *n* (%)	13 (72.2)	18 (81.8)	0.71
Age, mean (±SD)	61.4 (±17.7)	59.8 (±15.1)	0.76
DMARD naïve, *n* (%)	3 (16.7)	6 (27.3)	0.70
Biological use, *n* (%)	2 (11.1)	4 (18.2)	0.68
MTX use, *n* (%)	9 (50.0)	12 (54.5)	1.00
LEF use, *n* (%)	2 (11.1)	1 (4.5)	0.58
AZA use, *n* (%)	0 (0.0)	1 (4.5)	1.00
SSZ use, *n* (%)	1 (5.6)	1 (4.5)	1.00
HCQ use, *n* (%)	2 (11.1)	3 (13.6)	1.00
DAS28 Baseline, mean (±SD)	6.3 (±1.1)	5.2 (±1.5)	0.01

*p* values were calculated by Fisher exact or *t* test for, respectively, dichotomous and continuous variables

*AZA* azathioprine, *DAS28* disease activity score based on 28-joint count, *DMARD* disease-modifying anti-rheumatic drugs, *HCQ* hydroxychloroquine, *LEF* leflunomide, *MTX* methotrexate, *SSZ* sulfasalazine

Since concomitantly used disease-modifying anti-rheumatic drugs (DMARDs) might confound the investigated relationship of GCs and response, we additionally investigated the distribution of concomitant DMARDs use among the responders and non-responders (Online Resource 1). However, no effect of any DMARD on (non-)response was seen.

### Mutant allele frequencies

The observed allele frequency was lower than expected based on the general population; however, these differences did not reach statistical significance: 0.36 for T-allele of C1236T (0.43 expected, binominal test of proportion *p* = 0.10), 0.37 for A or T-allele of G2677AT (0.44 expected, *p* = 0.11), and 0.50 for T-allele C3435T (0.57 expected, *p* = 0.11).

### Genotype and response to GC therapy

The dominant model revealed an OR of 6.18 for G2677A/T polymorphism carriers and a trend for the other SNPs (Table 2). In the additive model, a trend for all SNPs and mutant allele carriers was found (data not shown). Stratified for the received treatment, wild-type carriers for all SNP in the IV cohort failed to achieve response, whereas 43–50% of the mutant allele carriers achieved a response (*p* = 0.05 for G2677A/T). For oral treatment, no differences in SNPs and response rates were observed.

**Table 2:** SNPs and clinical response to IV and oral GC treatment

SNP	Overall OR	*p* value for OR	Treatment	Genotype	Non-response, *n* (%)	Response, *n* (%)	*p* value
C1236T	1.96	0.35	IV	CC (*n* = 5)	5 (100)	0 (0)	0.11
				CT/TT (*n* = 13)	7 (54)	6 (46)	
			Oral	CC (*n* = 9)	5 (56)	4 (44)	0.66
				CT/TT (*n* = 13)	8 (62)	5 (39)	
G2677A/T	6.18	**0.04**	IV	GG (*n* = 6)	6 (100)	0 (0)	**0.05**
				GT/TT (*n* = 12)	6 (50)	6 (50)	
			Oral	GG (*n* = 8)	6 (75)	2 (25)	0.38
				GT/GA/AT/TT (*n* = 14)	7 (50)	7 (50)	
C3435T	1.69	0.51	IV	CC (*n* = 3)	3 (100)	0 (0)	0.52
				CT/TT (*n* = 15)	9 (60)	6 (40)	
			Oral	CC (*n* = 7)	4 (57)	3 (43)	1.00
				CT/TT (*n* = 15)	9 (60)	6 (40)	

Wild-type (for each respective allele) versus mutant allele carriers were compared in a logistic regression analysis corrected for treatment received. Per SNP the odds ratio (OR) and related *p* value are presented. In the subsequent columns, the absolute number of responders and non-responders per treatment is shown for wild-type and mutant allele carriers, including the overall *p* value (Fisher exact test). For the G2677AT polymorphism, there was a significant association between mutant allele carriage and response, though this association was restricted to the IV treated patients. Trends for the other SNPs and response to IV treatment were seen

### Functionality of P-gp

**Fig. 1:**
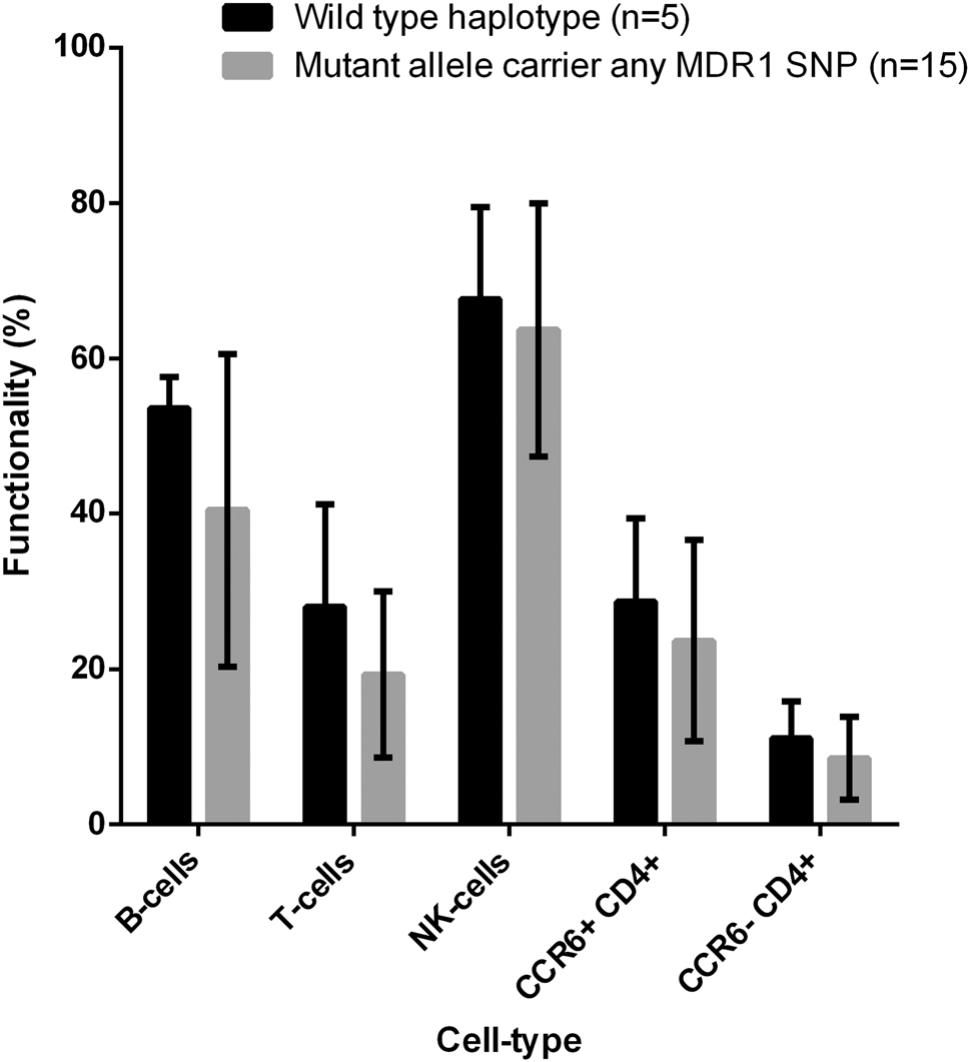
Functionality of P-gp for different subsets of PBMCs in %. Shown are the mean values and the standard deviations between wild-type carriers for all SNPs (haplotype carriers, *n* = 5) and mutant allele carriers for any MDR1 SNP (*n* = 15). CCR6+ CD4+ cells include most of the Th17 cells and subpopulations of regulatory- and memory T cells expressing CCR6+. Mutant allele carriers showed a decreased functionality of B cells (*t* test, *p* = 0.03) and minor decreased functionality for other cells subsets (all *p* > 0.10)

Of the originally 40 samples, 18 had to be excluded for P-gp functionality testing due to viability <40% and an additional two as they were taken after therapy initiation. Mutant allele carriers of all three SNPs showed lower functionality in B cells (*p* = 0.03), and for other cell sets, a small decrease in functionality was observed (all *p* > 0.10, Fig. 1).

## Discussion

This study investigated the role of polymorphisms in the MDR1 gene in the response to IV and oral GC therapy in RA, and showed that the response to IV methylprednisolone is significantly better in patients carrying the G2677A/T polymorphism compared to the wild-type carriers, with trends for the other SNPs. Mutant allele carriage for any MDR1 SNP was associated with a significant decreased P-gp functionality in B cells, whereas the impact on other PBMC subtypes was smaller but still present. Non-response to methylprednisolone might, therefore, be (partially) explained by the role of polymorphisms in the MDR1 gene and subsequent effect on P-gp activity.

The observed difference in effect of polymorphisms on the GC response in IV methylprednisolone compared to prednisone/prednisolone treated patients could be explained by two mechanisms. First, a profound difference in dependency on P-gp among GC analogues has been shown [14]. Methylprednisolone has the highest transport efficiency by P-gp of all GCs and thus is transported more efficiently than prednisolone (i.e. active component of prednisone). Consequently, an impaired function of P-gp due to a mutant allele exerts more impact in methylprednisolone compared with prednisone/prednisolone treated patients, which is in line with our results. Second, different substrate concentrations might lead to different effects on (dys)function. Whereas at low dose therapy (oral cohort), substrate influx and P-gp excretion may be in equilibrium, a drastic increase in substrate concentration, such as in IV pulse treatment, could result in a relative dysfunctionality of P-gp. This hypothesis fits the observations from in vitro work demonstrating differences in functionality between wild-type and polymorphism carriers at high substrate concentration (2.6 µM [15] and 5.0 µM [4]) but not at lower substrate concentrations (0.4 µM [16]). In this study, a substrate concentration of 2.6 µM was used, which showed lower efflux in B cells though smaller differences in other cell subsets.

Several other studies have focused on the relation of SNPs and response to GCs treatments in inflammatory diseases. Two studies investigated the C3435T polymorphism in RA and the effect on the combination therapy of methotrexate and GCs, which yielded better response rates for C3435T mutant allele carriers [17, 18]. Carriers of the G2677AT polymorphism in immune thrombocytopenia (ITP) have a better response to oral GCs, whereas the carriage of mutant alleles for C1236T and C3435T did not correlate with response [19]. In inflammatory bowel diseases, G2677AT did not relate to response, but C1236T and C3435T did [20]. The differences in results among these studies can partially be explained by factors related to demographics, disease, treatment, and study design (including statistical power of the different studies), and, therefore, do not exclude the possibility of a shared underlying pathway related to one or more SNPs.

Our study has its limitations with respect to the number of patients that were included, resulting in a reduced power for detecting effects and hampering correction for multiple testing. Therefore, the analyses should be considered exploratory, and, until validated in a bigger cohort, the results should be interpreted in that context. It should also be noted that although GC therapy was the subject of our study and concomitant treatments were stable, there were—albeit not significant—differences in used concomitant DMARDs between the cohorts and between responders and non-responders. Because of the low sample size, however, it was not possible to correct for these differences in multivariable analyses. In this study, the possibility of (extra-cytosolic) non-genomic effects to GC treatment was not considered. However, such effects are expected to appear more rapidly than 5/30 days and are, therefore, not likely to have influenced the observations. The chosen time intervals to evaluate the clinical response might have influenced the magnitude of the observed response, yet, the time points were based on empirical observations and were considered clinically most relevant. Another limitation is the low viability of PBMCs after thawing, possibly related to factors, such as transport or the age of the cells. The dead cells could have disturbed the results of the functionality assay, which would explain the limited differences in P-gp functionality between the polymorphisms.

Our data suggest that RA-patients with wild-type polymorphisms could potentially benefit from concomitant MDR1-inhibition (such as by cyclosporine, tacrolimus, and hydroxychloroquine [3]), administration of GC analogues that are less sensitive to P-gp transport (or not a substrate for P-gp), or treatments that are not influenced by P-gp, e.g., extracellular acting treatments, such as tumor necrosis factor alpha inhibitors. Additional work needs to be undertaken using larger patient cohorts to better clarify the association between MDR1 SNPs, P-gp activity and response to GCs.

## Electronic supplementary material

Below is the link to the electronic supplementary material.

Concomitant treatments among responders and non-responders to GC therapy (XLSX 9 KB)

Original data supporting the findings of this research. Within this data file, per patient, the treatment, clinical response, genotyping, and functionality of P-gp can be found (XLSX 16 KB)

## References

[1] Kirwan JR, Bijlsma JW, Boers M, Shea BJ (2007). Effects of glucocorticoids on radiological progression in rheumatoid arthritis. Cochrane Database Syst Rev.

[2] Hoffmeyer S, Burk O, von RO, Arnold HP, Brockmoller J, Johne A, Cascorbi I, Gerloff T, Roots I, Eichelbaum M, Brinkmann U (2000). Functional polymorphisms of the human multidrug-resistance gene: multiple sequence variations and correlation of one allele with P-glycoprotein expression and activity in vivo. Proc Natl Acad Sci USA.

[3] Tsujimura S, Tanaka Y (2015). Disease control by regulation of P-glycoprotein on lymphocytes in patients with rheumatoid arthritis. World. J Exp Med.

[4] Salama NN, Yang Z, Bui T, Ho RJ (2006). MDR1 haplotypes significantly minimize intracellular uptake and transcellular P-gp substrate transport in recombinant LLC-PK1 cells. J Pharm Sci.

[5] Tsujimura S, Saito K, Nawata M, Nakayamada S, Tanaka Y (2008). Overcoming drug resistance induced by P-glycoprotein on lymphocytes in patients with refractory rheumatoid arthritis. Ann Rheum Dis.

[6] Ueda K, Okamura N, Hirai M, Tanigawara Y, Saeki T, Kioka N, Komano T, Hori R (1992). Human P-glycoprotein transports cortisol, aldosterone, and dexamethasone, but not progesterone. J Biol Chem.

[7] Webster JI, Carlstedt-Duke J (2002). Involvement of multidrug resistance proteins (MDR) in the modulation of glucocorticoid response. J Steroid Biochem Mol Biol.

[8] Jacobs JW, Geenen R, Evers AW, van Jaarsveld CH, Kraaimaat FW, Bijlsma JW (2001). Short term effects of corticosteroid pulse treatment on disease activity and the wellbeing of patients with active rheumatoid arthritis. Ann Rheum Dis.

[9] Forster PJ, Grindulis KA, Neumann V, Hubball S, McConkey B (1982). High-dose intravenous methylprednisolone in rheumatoid arthritis. Ann Rheum Dis.

[10] de Jong PH, Hazes JM, Barendregt PJ, Huisman M, van Zeben D, van der Lubbe PA, Gerards AH, de Jager MH, de Sonnaville PB, Grillet BA, Luime JJ, Weel AE (2013). Induction therapy with a combination of DMARDs is better than methotrexate monotherapy: first results of the tREACH trial. Ann Rheum Dis.

[11] de Jong PH, Quax RA, Huisman M, Gerards AH, Feelders RA, de Sonnaville PB, Luime JJ, Weel AE, Hazes JM (2013). Response to glucocorticoids at 2weeks predicts the effectiveness of DMARD induction therapy at 3months: post hoc analyses from the tREACH study. Ann Rheum Dis.

[12] van Gestel AM, Haagsma CJ, van Riel PL (1998). Validation of rheumatoid arthritis improvement criteria that include simplified joint counts. Arthritis Rheum.

[13] Institute TEB (2015) 1000 Genomes; A Deep Catalog of Human Genetic Variation. http://www.1000genomes.org/; Accessed: 23 July 2015

[14] Yates CR, Chang C, Kearbey JD, Yasuda K, Schuetz EG, Miller DD, Dalton JT, Swaan PW (2003). Structural determinants of P-glycoprotein-mediated transport of glucocorticoids. Pharm Res.

[15] Hitzl M, Drescher S, van der Kuip H, Schaffeler E, Fischer J, Schwab M, Eichelbaum M, Fromm MF (2001). The C3435T mutation in the human MDR1 gene is associated with altered efflux of the P-glycoprotein substrate rhodamine 123 from CD56 + natural killer cells. Pharmacogenetics.

[16] Oselin K, Gerloff T, Mrozikiewicz PM, Pahkla R, Roots I (2003). MDR1 polymorphisms G2677T in exon 21 and C3435T in exon 26 fail to affect rhodamine 123 efflux in peripheral blood lymphocytes. Fundam Clin Pharmacol.

[17] Pawlik A, Wrzesniewska J, Fiedorowicz-Fabrycy I, Gawronska-Szklarz B (2004). The MDR1 3435 polymorphism in patients with rheumatoid arthritis. Int J Clin Pharmacol Ther.

[18] Drozdzik M, Rudas T, Pawlik A, Kurzawski M, Czerny B, Gornik W, Herczynska M (2006). The effect of 3435C > T MDR1 gene polymorphism on rheumatoid arthritis treatment with disease-modifying antirheumatic drugs. Eur J Clin Pharmacol.

[19] Xuan M, Li H, Fu R, Yang Y, Zhang D, Zhang X, Yang R (2014). Association of ABCB1 gene polymorphisms and haplotypes with therapeutic efficacy of glucocorticoids in Chinese patients with immune thrombocytopenia. Hum Immunol.

[20] Yang QF, Chen BL, Zhang QS, Zhu ZH, Hu B, He Y, Gao X, Wang YM, Hu PJ, Chen MH, Zeng ZR (2015). Contribution of MDR1 gene polymorphisms on IBD predisposition and response to glucocorticoids in IBD in a Chinese population. J Dig Dis.

